# Evaluating the utility of inflammatory markers in the diagnosis of soft tissue abscesses of the forearm and hand

**DOI:** 10.5194/jbji-8-119-2023

**Published:** 2023-03-30

**Authors:** Sarah R. Blumenthal, Adnan N. Cheema, Steven E. Zhang, Benjamin L. Gray, Nikolas H. Kazmers

**Affiliations:** 1 Department of Orthopaedic Surgery, University of Pennsylvania, Philadelphia, PA 19104, USA; 2 Department of Orthopaedic Surgery, University of Utah, Salt Lake City, UT 84108, USA

## Abstract

Upper extremity abscesses frequently present to the acute care setting with inconclusive physical examination and imaging findings. We sought to investigate the diagnostic accuracy of inflammatory markers including white blood cell (WBC) count, erythrocyte sedimentation rate (ESR), and C-reactive protein (CRP). A retrospective cohort study was performed to identify subjects ≥18 years treated with surgical debridement of upper extremity abscesses at our institution between January 2012 and December 2015. In this study, 188 patients were screened, and 72 met the inclusion criteria. A confirmed abscess as defined by culture positivity was present in 67 (93.1 %) cases. The sensitivity of WBC, ESR, or CRP individually was 0.45, 0.71, and 0.81. The specificity of WBC, ESR, or CRP individually was 0.80, 0.80, and 0.40. In combination all three markers when positive had a sensitivity of 0.26 and specificity of 1.0. These values were similar among patients with diabetes and those with obesity. With the highest sensitivity and lowest specificity, CRP exhibited the most utility as a screening test (level IV).

## 1 Introduction

Upper extremity soft tissue infections encompass a broad variety of clinical conditions, ranging from cellulitis to deep abscesses, which may pose a diagnostic challenge for the surgeon recommending operative versus non-operative management (McDonald et al., 2011; Rigopoulos et al., 2012). The upper extremity is one of the most common locations of soft tissue infections presenting to the emergency department, and the incidence continues to rise (Taira et al., 2009). The severity of these infections ranges from cellulitis, manageable with intravenous antibiotics, to deep abscesses that require urgent debridement. A surgeon's index of suspicion is generally based primarily on clinical examination, and objective validated measures of surgical indications are often lacking. Currently, advanced imaging in conjunction with laboratory values including white blood cell (WBC) count, C-reactive protein (CRP), and erythrocyte sedimentation rate (ESR) are the only objective measures that alert clinicians to the presence of underlying pathology. These acute phase proteins and immune response markers rise early in response to physiological insult or stress, as in the case of hand infection, but they also may be elevated secondary to other concurrent diseases (Fogler and Lindsey, 1998; Litao and Kamat, 2014). Therefore, their interpretations must be made with the entire clinical picture in mind.

A classic example of a well-defined algorithm in evaluating likelihood of infectious etiology is the Kocher criteria, which differentiate between septic arthritis of the hip and transient synovitis in a child who presents with an acutely painful hip. With all four clinical signs present – elevated WBC, increased ESR, unwillingness to bear weight, and fever – a patient has a 99 % likelihood of having a septic hip (Kocher et al., 1999). Attempts have been made to apply similar algorithms to other infectious processes without achieving the same level of validation (Singhal et al., 2011; Sultan and Hughes, 2010). Bishop et al. (2013) endeavored to generate a similar algorithm for purulent flexor tenosynovitis, but such an evaluation in the realm of upper extremity abscesses specifically has not been published to our knowledge. Further, currently there are conflicting data on the value of each aforementioned marker. Early data suggested that ESR might be the best test for hand infections, but more recent research appears to substantiate CRP as a more reliable indicator (Gauger et al., 2021; Houshian et al., 2006). No studies to date have evaluated their use specifically in the diagnosis of upper extremity abscess.

Given the paucity of data on the utility of these laboratory values in applicable treatment algorithms, we sought to evaluate whether inflammatory markers, specifically WBC, ESR, and CRP, can reliably be applied in an emergency setting to identify abscesses of the upper extremity.

## 2 Methods

**Figure 1:**
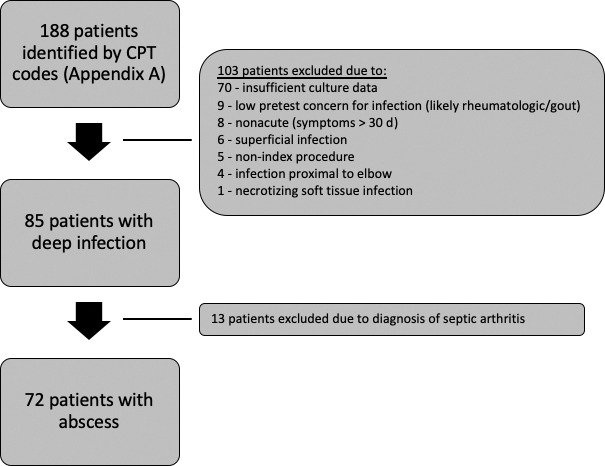
Exclusion criteria applied to obtain the final study cohort.

Institutional Review Board (IRB) approval was obtained for this retrospective cohort study. In total, 188 patients treated with surgical debridement for acute abscesses of the upper extremity at an urban academic medical center were reviewed. An acute infection was defined as that with symptoms less than 30 d. Patients greater than 18 years who underwent irrigation and debridement in the operating room between December 2012 and October 2015 were included. They were identified using Current Procedural Terminology (CPT) codes specific for the treatment of acute abscesses (Supplement). Patients with abscesses proximal to the elbow were excluded. Patients with other concomitant pathologies such as rheumatologic disorders or coexisting infectious processes such as suppurative flexor tenosynovitis, septic arthritis, osteomyelitis, necrotizing fascitis, and septic bursitis were also excluded (Fig. 1) due to concerns for inconsistent diagnostic coding and frequent use of multiple codes in these instances. Charts of all patients identified by CPT codes were manually reviewed to determine whether inclusion criteria were met.

Intraoperative culture results including aerobic and anaerobic bacterial, mycobacterial, and fungal cultures were reviewed to confirm that fluid was sent for microbiology analysis. Any patients without documentation of these cultures, positive or negative, were excluded. For patients who underwent multiple surgical debridements, only cultures from the index procedure were included. Cultures obtained in the emergency department or outside the intraoperative setting were excluded. All patient electronic medical records were reviewed for the documentation of WBC, ESR, and CRP within 24 h of presentation. Documentation of at least one of these three inflammatory markers was required for inclusion. Laboratory cutoffs for normal values at our institution were WBC ≤11.0 , ESR ≤15 , and CRP (non-cardiac) ≤0.80 .

Demographics, clinical history, operative notes, and cultures were reviewed for each patient to ensure appropriate diagnosis for inclusion. Demographic data that were collected included age, sex, body mass index (BMI), and comorbidities, specifically diabetes mellitus and obesity. Obesity was defined as BMI ≥30 (NHLBI, 1998).

The positivity of any cultures was used as the gold standard to identify patients with true abscesses. Notations and descriptions of purulence in operative reports were reviewed, but ultimately reporting was too inconsistent to be deemed appropriate for inclusion. Sensitivity, specificity, positive predictive value (PPV), and negative predictive value (NPV) were calculated for WBC, ESR, and CRP in comparison to true positive cases. Calculations for each individual marker were performed within the subgroup for which those laboratory tests were drawn. Factors that might affect susceptibility to infection such as diabetes mellitus and obesity were then compared to the presence of positive cultures. Sensitivity, specificity, PPV, and NPV were also calculated for “all markers positive” if all three were elevated and “any marker positive” if at least one marker was elevated.

## 3 Results

In this study, 188 patients were screened, with 72 patients meeting inclusion criteria for pre-operative diagnosis of an upper extremity abscess. Of patients included, 63 (58 %) were male. The average age was 47.8 (SD of 14.5) (Table 1). Among all study patients, 65 aerobic cultures (90.3 %), 23 (31.9 %) anaerobic cultures, and 3 (4.2 %) fungal cultures were positive. A true abscess as defined by any culture positivity was present in 67 (93.1 %) of cases. True positives and true negatives varied by inflammatory marker and were calculated both separately and in combination when all were elevated.

**Table 1:** Patient characteristics for subjects who met inclusion criteria ( n=72 ).

Patient characteristics	
Total patients	72
Average age (years)	47.8 (SD of 14.5)
Male	58 %
Female	42 %
Diabetes	26 %
Obesity	31 %

WBC count was present and documented for all patients. It was elevated in 31 patients (43.0 %). CRP was documented in 57 patients and was elevated in 78.9 % of documented cases. ESR was documented in 53 patients and was elevated in 66.0 % of cases. All three markers were documented for 52 patients, or 72.2 % of the total study cohort.

**Figure 2:**
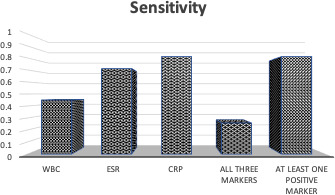
Sensitivity for true abscess when either WBC, ESR, CRP, all three markers, or at least one marker were positive. In order, the sensitivities were 0.45, 0.71, 0.81, 0.26, and 0.81.

**Figure 3:**
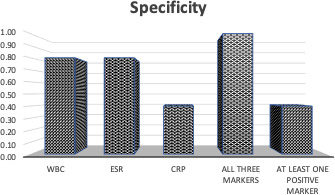
Specificity for true abscess when either WBC, ESR, CRP, all three markers, or at least one marker were positive. In order, the specificities were 0.80, 0.80, 0.40, 1.00, and 0.40.

A positive CRP alone demonstrated higher sensitivity than ESR alone (0.81 versus 0.71), while a positive WBC demonstrated the lowest sensitivity of 0.45 (Fig. 2). When at least one inflammatory marker (ESR, CRP, or WBC) was positive, sensitivity was 0.81. WBC and ESR showed equal specificities of 0.80, while CRP had the lowest specificity of 0.40 (Fig. 3). When all three inflammatory markers were positive, sensitivity decreased to 0.26, but the specificity rose to 1.0.

Positive predictive values (PPVs) were high across all markers. PPV for either a positive ESR or WBC was the highest at 0.97. A positive CRP had a PPV of 0.93. When all inflammatory markers were positive, the PPV was 1.0 and only fell to 0.95 if any one marker was positive. In contrast, the negative predictive values (NPVs) of these markers were low overall. ESR had the highest NPV of 0.22, followed by CRP (0.17) and WBC (0.10). The NPV was 0.13 for all three markers positive and for at least one marker positive.

For patients with diabetes mellitus ( n=19 ), sensitivity was 0.29 when all markers of infection were positive (compared to 0.26 when analyzing all patients), while specificity remained at 1.0. Sensitivities of WBC, ESR, and CRP were 0.44, 0.87, and 0.80; specificities were 1.0, 1.0, and 0. For patients whose body mass index (BMI) met the threshold for obesity at ≥30 ( n=25 ), the sensitivity of all markers positive was 0.17 and the specificity was 1.0. Sensitivities of WBC, ESR, and CRP for obese patients were 0.43, 0.72, and 0.85; specificities were 1.0, 1.0, and 0.5.

## 4 Discussion

Upper extremity infections are common, particularly in acute care settings (Fowler and Ilyas, 2013). Clinicians must consider a broad range of differential diagnoses when evaluating a painful, swollen, and erythematous upper extremity. Making the diagnosis can be difficult and relies on a combination of clinical judgment, imaging, and laboratory testing. The weight assigned to each of these factors is subjective and varies according to the practitioner, with insufficient literature to validate a standardized approach in establishing the diagnosis of upper extremity abscesses. Furthermore, early detection of these infections is integral to treatment, as the time to surgery or antibiosis is associated with improved outcomes and lower complications (Glass, 1982; Osterman et al., 2014). Objective imaging such as ultrasound, CT, and MRI findings may be used to clarify the clinical picture but may not always be available in community emergency settings. In such instances, laboratory data may be used to support but not supplant clinical judgment. The utility of these findings may vary based on the availability of the abovementioned imaging tests.

We were unable to identify any prior literature that evaluates the role of inflammatory markers as a screening test in the identification of upper extremity abscesses. To our knowledge, only two studies to date have examined the use of inflammatory markers in the diagnosis of upper extremity infections. In 2013, Bishop et al. (2013) reported on 82 patients with purulent flexor tenosynovitis, of which 71 underwent urgent surgical debridement. The authors found that all three markers had a specificity and positive predictive value of 1.0. Similar to our data, they found that CRP was most sensitive at 0.76, with ESR and CRP nearly identical at 0.41 and 0.39, respectively. More recently in 2019, Gauger et al. (2019) published a similar retrospective investigation that evaluated WBC, ESR, and CRP in 61 patients with an upper extremity infection requiring operative debridement. They more broadly included all infections without excluding specific diagnoses or sub-categorizing results by diagnosis. They found CRP to be the most sensitive test, as it was elevated in 90 % of culture-positive patients, compared to WBC at 54 % and ESR at 67 %.

Together, our data indicate that the presence of elevated inflammatory markers may be used as an adjunct tool in settings of high clinical suspicion with an equivocal exam or imaging to aid in confirming the diagnosis of upper extremity abscesses. The PPV for each of the three inflammatory markers in our study was consistently high and improved to 1.0 when all three markers were positive. The generally high positive predictive values and low negative predictive values that we identified are consistent with those of Bishop's group. They found that while negative inflammatory markers could not be used reliably to rule out infection, positive values allowed clinicians to confirm the presence of flexor tenosynovitis in a setting of high clinical suspicion. Of note, unlike Bishop's group, we found a lower specificity for CRP (0.40 compared to 1.0), suggesting that its use in isolation might lead to an overestimation of patients with true upper extremity abscesses. This discrepancy also implies that the inflammatory response elicited by flexor tenosynovitis and abscesses, at least in terms of CRP levels, may differ. This detail highlights the importance of understanding the pathogenesis behind different inflammatory markers in various infection subtypes. For this reason, we also elected to narrow our criteria for inclusion rather than compare infections resulting from other pathologic classifications.

In our study, the specificity of all three markers in combination when positive was 1.0 and stayed at 1.0 regardless of the presence of diabetes mellitus or obesity. CRP had high sensitivities in each group at 0.81 in all patients, 0.80 in patients with diabetes mellitus, and 0.85 in patients with obesity. Of note, ESR had a higher sensitivity in patients with diabetes at 0.87, which may represent the baseline chronic inflammatory milieu of the disease process. These results hold considerable clinical importance for those patients at higher risk for infection and with more devastating complications of inadequate treatment, as patients with diabetes may have up to a 39 % chance of amputation after developing an upper extremity infection that requires operative debridement (Gonzalez et al., 1999). Prior research has demonstrated that patients with diabetes have elevated baseline ESR and CRP compared to non-diabetics, yet WBC and CRP recently have been shown to be comparable in diabetics and non-diabetics with hand infections (Hayden et al., 2020). For diabetic hand infections involving an abscess, prompt recognition followed by timely surgical debridement and initiation of appropriate antibiosis is imperative (Jalil et al., 2011).

Our study had several limitations. First, this study was conducted at a single tertiary care center with procedures performed by three hand surgeons. The population also was limited to a single geographic area with high referral rates. It may be possible that a higher percentage of patients who present to this institution have more severe upper extremity infections when compared to the general population. Furthermore, our study is limited by its retrospective nature and may suffer from selection bias, as only patients who were deemed high risk for abscesses and who underwent surgical debridement were included. This could have contributed to the low negative predictive values we found. As there was no control group, it was not possible to calculate likelihood ratios. We are also aware that laboratory reference cutoffs may differ between centers, and as such we used binary metrics to represent increased values rather than magnitude of elevation. Future research is needed to clarify any significance to varying degrees of laboratory value elevation. Finally, our inclusion of patients with at least one inflammatory marker documented likely underestimates the number of patients with elevated markers which might lead to higher sensitivities. Nevertheless, we believe that these limitations do not detract from the conclusions of this study.

## 5 Conclusions

Inflammatory markers are routinely used to help diagnose upper extremity infections and more specifically abscesses that require surgical debridement. In isolation, an elevation of CRP and ESR is associated with relatively high sensitivity for the presence of an abscess. Further investigation is needed to better elucidate the weight assigned to each variable. Although laboratory markers are not sufficient to supplant clinical judgment and objective imaging data, they may supplement and support other findings in the appropriate clinical context. Nevertheless, elevation of these three markers may be used reliably to substantiate the diagnosis of an upper extremity abscess requiring surgical intervention, in particular in community settings without readily available advanced imaging.

## Supplement

10.5194/jbji-8-119-2023-supplement The supplement related to this article is available online at: https://doi.org/10.5194/jbji-8-119-2023-supplement.

## Data Availability

Data are not publicly available due to Institutional Review Board policies on human subjects.
