# Supplementary material for: Evaluating the utility of inflammatory markers in the diagnosis of soft tissue abscesses of the forearm and hand
Source: J Bone Jt Infect. 2023 Mar 30;8(2):119–23. doi: 10.5194/jbji-8-119-2023 (PMC10077572; doi:10.5194/jbji-8-119-2023)
Supplement: The supplement related to this article is available online at: https://doi.org/10.5194/jbji-8-119-2023-supplement. [file jbji-8-119-supplement.pdf]

Supplement of J. Bone Joint Infect., 8, 119–123, 2023
https://doi.org/10.5194/jbji-8-119-2023-supplement
© Author(s) 2023. CC BY 4.0 License.

JBJI
Journal of Bone and Joint Infection
Open Access

Supplement of

Evaluating the utility of inflammatory markers in the diagnosis of soft tissue abscesses of the forearm and hand

Sarah R. Blumenthal et al.

Correspondence to: Sarah R. Blumenthal (sarahrblumenthal@gmail.com)

The copyright of individual parts of the supplement might differ from the article licence.

Supplement: Current Procedural Terminology (CPT) codes used to identify patients for study inclusion, followed by manual review

10060 - Incision and drainage of abscess eg, carbuncle, suppurative hidradenitis, and other cutaneous or subcutaneous abscess, cyst, furuncle , or paronychia; simple or single

10061 - Incision and drainage of abscess eg, carbuncle, suppurative hidradenitis, and other cutaneous or subcutaneous abscess, cyst, furuncle, or paronychia); complicated or multiple

10140 - Incision and drainage of hematoma, seroma or fluid collection

10180 - Incision and drainage, complex, postoperative wound infection

20000 - Incision of soft tissue abscess eg, secondary to osteomyelitis) ; superficial

20005 - Incision of soft tissue abscess eg, secondary to osteomyelitis) ; deep or complicated

23930 - Incision deep, with opening of bone cortex; elbow or upper arm

23931 - Incision and drainage elbow bursa

25028 - Incision and drainage, forearm and/or wrist; deep abscess or hematoma

25031 - Incision and drainage, forearm and/or wrist; infected bursa

25035 - Incision, deep, with opening of bone cortex eg, for osteomyelitis or bone abscess), forearm and/or wrist

25040 - Arthrotomy, radiocarpal or mediocarpal joint, with exploration, drainage, or removal of foreign body

26010 - Drainage of finger abscess; simple

26011 - Drainage of finger abscess; complicated eg, felon, etc)

26020 - Drainage of tendon sheath, one digit and/or palm

26025 - Drainage of palmar bursa; single, ulnar or radial

26030 - Drainage of palmar bursa; multiple or complicated

26034 - Incision, deep, with opening of bone cortex eg, for osteomyelitis or bone abscess), hand or finger

26070 - Arthrotomy, for infection, with exploration, drainage or removal of foreign body; carpometacarpal joint

26075 - Arthrotomy, for infection, with exploration, drainage or removal of foreign body; metacarpophalangeal joint

26080 - Arthrotomy, for infection, with exploration, drainage or removal of foreign body; interphalangeal joint, each
